# The Trypanosome Pumilio Domain Protein PUF5

**DOI:** 10.1371/journal.pone.0077371

**Published:** 2013-10-22

**Authors:** Bhaskar Anand Jha, Stuart K. Archer, Christine E. Clayton

**Affiliations:** Zentrum für Molekulare Biologie der Universität Heidelberg, DKFZ-ZMBH Allianz, Heidelberg, Germany; University of Texas Medical School at Houston, United States of America

## Abstract

PUF proteins are a conserved family of RNA binding proteins found in all eukaryotes examined so far. This study focussed on PUF5, one of 11 PUF family members encoded in the *Trypanosoma brucei* genome. Native PUF5 is present at less than 50000 molecules per cell in both bloodstream and procyclic form trypanosomes. C-terminally myc-tagged PUF5 was mainly found in the cytoplasm and could be cross-linked to RNA. PUF5 knockdown by RNA interference had no effect on the growth of bloodstream forms. Procyclic forms lacking PUF5 grew normally, but expression of PUF5 bearing a 21 kDa tandem affinity purification tag inhibited growth. Knockdown of *PUF5* did not have any effect on the ability of trypanosomes to differentiate from the mammalian to the insect form of the parasite.

## Introduction

PUF proteins were named after the two founding members: Pumilio in *Drosophila melanogaster* and FBF in *Caenorhabditis elegans*. They contain up to 8 copies of a nucleotide-binding repeat in their RNA binding ‘PUF’ domain [1] which recognises sequences that include the motif UGUR [2], [3], [4], [5]. PUF proteins play various roles in RNA metabolism. Although some have roles in ribosomal RNA synthesis and processing [6], [7], the majority of publications focus on the ability of PUF proteins to destabilise, or repress translation of, target mRNAs [8], [9], [10]. The cytoplasmic *Saccharomyces cerevisiae* PUF proteins are not required for growth on rich media; indeed, *S. cerevisiae* strains lacking all five cytoplasmic PUF proteins Puf1–5 are viable, despite showing differences in the abundances of some transcripts [11]. Yeast Puf3p destabilizes some mitochondrion-related transcripts, depending on the available carbon source, while Puf4p is involved in the regulation of stability of mRNAs coding for ribosomal proteins and ribosome biogenesis factors in response to starvation and heat shock [12]. Several other results suggest that PUF proteins can have redundant functions. *S. cerevisiae* Puf4p and Mpt5p both regulate the expression of mRNA encoding the HO endonuclease [13]; and Puf1p and Puf5p can individually stimulate the decay of *TIF1* mRNA but both proteins are needed for full regulation [14]. Similarly, *C. elegans* FBF-1 and FBF-2 are redundant in controlling mitosis [15]. In HeLa S3 cancer cells, PUM1 and PUM2 have numerous overlapping targets and were shown to potentially regulate approximately 15% of the cell’s transcriptome [16]. Finally, yeast PUF proteins can influence mRNA localization: Puf3p localizes its target mRNAs to mitochondria [17], and Puf5p has role in the localization of *PEX14* mRNA near peroxisomes [18].

*Trypanosoma brucei* is the causative agent of Human African Trypanosomiasis or sleeping sickness [19]. Trypanosomes are unicellular parasites of the order Kinetoplastida. Bloodstream forms grow in mammalian hosts and are transmitted by Tsetse flies [20]; the form that grows in the fly mid-gut is called “procyclic”. Transcription of most protein-coding genes in trypanosomes is polycistronic, with no evidence for developmental regulation of initiation [21]. Gene expression is therefore mainly controlled at the levels of mRNA turnover and translation [22]. In both trypanosomes [22], [23] and other eukaryotes [24], RNA binding proteins play a major role in this regulation.

The genome of *Trypanosoma brucei* encodes over 150 RNA binding proteins, including at least 11 Pumilio domain proteins [25], [26]. Of these, *Tb*PUF7 is related to yeast Nop9p, and is involved rRNA processing [6]; and *Tb*PUF8 is related to yeast Puf6p, another nucleolar factor [6] (although Puf6p was also reported to influence *ASH1* mRNA translation [27]). *Tb*PUF10 is also in the nucleolus, and influences not only rRNA processing, the expression of GPEET procyclin in procyclic forms [28]. *T. brucei* PUF9 was the first Puf-domain protein to be shown to stabilize, rather than destabilize target transcripts: it is specific for a few mRNAs that increase during the S phase of the cell cycle [29]. *Tb*PUF1 is not essential for parasite survival [26]. It binds to repetitive and transposon-type elements but a knock-out had no detectable effects on either the transcriptome or the proteome [26]. In contrast, *Tc*PUF6, the *T. cruzi* homologue of *Tb*PUF1, was shown to bind to several mRNAs, some of which were decreased by *Tc*PUF6 over-expression [30]. The latter result must, however, be interpreted with caution since over-expression of RNA-binding proteins can result in their binding to RNAs which are not normally targets.

In our initial experiments, RNAi targeting the remaining *T. brucei* PUF proteins (from now on written without the *Tb* prefix) had no effect on growth of bloodstream and procyclic trypanosomes [26]. However, in a high-throughput RNAi screen, Alsford *et al.* found that *PUF5* RNAi was deleterious in cells undergoing differentiation. Moreover, they also found that depletion of some other trypanosome PUFs (PUF2, PUF9 and PUF4) affected trypanosome growth [31]. This study focuses on the role of PUF5 in bloodstream and procyclic forms and during differentiation.

## Methods

### Plasmid Constructs

Plasmids and oligonucleotides are listed in File S1 as Tables S1 and S2 respectively. To express tagged PUF5 (*Tb*927.7.4730), the open reading frame (ORF) was amplified (primers CZ2259 and CZ2261). For C-terminal tandem affinity purification (TAP) and myc tagging, the *PUF5* PCR product was cloned in the vectors pHD918 [32] and pHD1700 [33] respectively, giving pHD2176 (TAP tag) and pHD1788 (2xmyc tag).

For RNAi against *PUF5*, a 436 bp fragment of the ORF from pHD1652 [26] was inserted into p2T7^TA^-blu [34]. For RNAi in pleomorphic EATRO 1125 cells, a stem-loop RNAi construct (pHD2178) was prepared using the primers CZ3956 and CZ3957 [35].

To make knockout vectors pBlueScript SK-based plasmids pHD1747 (puromycin resistance) and pHD1748 (blasticidin resistance) were used. The knockout plasmids contained a 275 bp fragment of the 5′ UTR (primers CZ2749 and CZ2750) and a 272 bp fragment of the 3′ UTR (primers CZ2755 and CZ2732) (pHD2385 and pHD2179).

### Trypanosome Culture

Trypanosomes (Lister 427 strain expressing the tet repressor from pHD1313) were cultured and transfected as described [34] and the expression of tetracycline inducible genes was induced using 100 ng/ml of tetracycline (tet) [35]. Cells were diluted to a density of 5×10^5^ cells/ml (procyclic) 1×10^5^ cells/ml (bloodstream) as required.

EATRO 1125 trypanosomes were cultured as previously described [36], [37] and transfected with the plasmid pHD2178 the knockdown plasmid for *PUF5*. RNAi was induced at a density of 2×105cells/ml. Differentiation was induced 24 h later (density 1–2×106cells/ml) by the addition of cis-aconitate (6 mM) and transferring the culture to 27°C [38]. After 24 hours the cells were pelleted and resuspended in procyclic media at approximately 3×10^6^cells/ ml.

### Antibody Production and Affinity Purification

The complete ORF of *PUF5* was PCR amplified, and cloned in the pQTEV-based (Addgene, Cambridge, USA) vector pHD1746 (10×His tags), to give pHD2153. *E. coli DH5α* cells were transformed and His-tagged protein was purified using Ni-NTA superflow resin (Qiagen, Germany). Recombinant PUF5 was insoluble, so it could not be used for *in vitro* binding studies. Antibody was raised in a rabbit against the purified PUF5 protein (Charles River Laboratories, Kisslegg, Germany). The serum was affinity purified using His-tagged PUF5 bound to nitrocellulose [39]. To check sensitivity, 20–200 ng of the recombinant protein were loaded on a 10% SDS-PAGE and the blot was probed with 1∶500 and 1∶2000 dilutions of the purified antibody.

### Immunofluorescence

Cells were harvested 24 hours after induction of PUF5-myc expression. Primary anti-myc antibody (Santa Cruz Biotechnology, Germany) and secondary anti-mouse antibody coupled to Alexa 488 (Life technologies, Germany) were used for indirect immunofluorescence as described [40]. Z-stacks of the cells were taken using the Olympus Cell-R microscope and the images thus obtained were deconvoluted using the Cell-R software (Wiener deconvolution algorithm).

### Northern Blotting and PCR for Knockout Verification

Trypanosomes in the log phase of growth (0.8–1.2×10^6^ cells/ml for bloodstream forms and 2–3×10^6^cells/ml for procyclics) were harvested. RNA was prepared using Trifast (Peqlab, Germany). 10 µg of total RNA was separated on a denaturing formaldehyde agarose gel followed by blotting and hybridization with ^32^P labelled probe (PCR using the primers CZ2262 and CZ2263). The radioactive signals were detected on phosphorimager (FLA 3000, Fujifilm).

For knockout cell line verification, PCR was done using the primers CZ3956 and CZ3957 in the *PUF5* ORF. The primers CZ4024 and CZ4025, generating a 708 bp fragment of *TbPUF2* ORF, served as positive control.

### Crosslinking and Immunoprecipitation (CLIP)

To check the RNA binding ability of PUF5, around 1×10^9^ procyclic cells inducibly expressing PUF5-myc, C-terminally myc tagged UBP1 (UBP1-myc) [41] or no tagged protein (wild type) were harvested after 24 hours of tet induction. All the cultures had a density of ≈3.0×10^6^ cells/ml. All cultures were cross-linked twice using 0.4 J/cm^2^ 254 nm UV light in a Stratalinker 2400, with mixing in between. The cells were then immediately pelleted at 4°C followed by washing with ice cold PBS. The pellets were snap frozen in liquid nitrogen and stored at −80°C. [36], [42], [43] with a few changes. Tagged proteins were immunoprecipitated using the anti-c-myc antibody-coupled agarose beads (Bethyl laboratories, TX, USA). RNA-protein complexes bound to the beads were digested with RNase T1 (Fermentas, Germany) (10 U/µl of the original bead volume). Residual RNAs were dephosphorylated and 5′ end labelled by ^32^P, then the preparation was run on a 4–12% Novex NuPAGE Bis-Tris gel (Invitrogen, Germany). Saved aliquots of approximately 10% from the lysate, unbound and bound fractions were used for western blotting to check the efficiency of immunoprecipitation.

## Results and Discussion

### PUF5 is in the Cytoplasm and Present at Less than 5×10^4^ Molecules Per Cell

**Figure 1:**
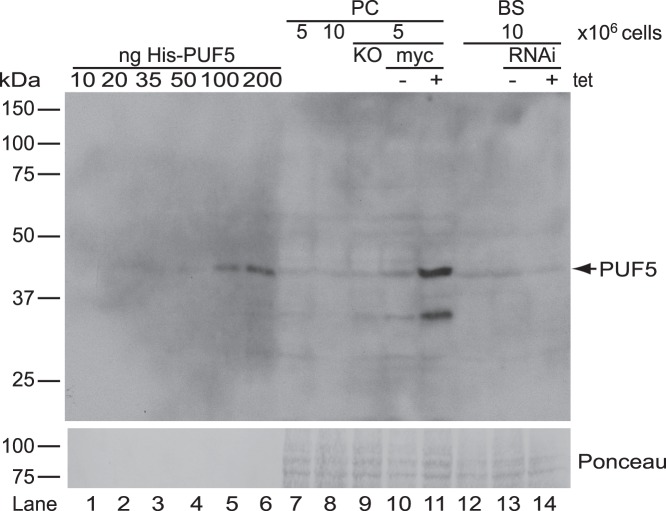
Endogenous PUF5 is not detected by a specific polyclonal antibody. Affinity purified anti-PUF5 antibody does not detect PUF5 in bloodstream-form (BS) and procyclic-form (PC) trypanosomes. Upper panel: From lanes 1 to 6, increasing amounts of recombinant protein were loaded. From lanes 7 to 11 and lanes 12 to 14, PC and BS cells were loaded, respectively. The very faint band that co-migrates with PUF5 in lanes 7, 8, 12 and 13 was background since it was also seen for the knockout (lane 9) and induced RNAi (lane 14). Lower panel: Ponceau S stain of the blot as a loading control.

To check the expression of PUF5 in bloodstream and procyclic cells, the protein was His-tagged and purified from *E. coli*. A polyclonal antibody raised against this protein was just able to detect 50 ng recombinant PUF5 (Fig. 1, lane 4∶6.25×10^11^ molecules) and easily detects 100 ng. Since no clear PUF5 signal was obtained from either 1×10^7^ PC or BS trypanosomes (Fig. 1, lanes 8 and 12), there are probably less than 5×10^4^ molecules of PUF5 protein per cell. How much lower expression is, we cannot tell. The measurement in trypanosome extracts was made more difficult by a faint cross-reacting band at roughly the same place in the gel which was also seen in the knock-out line (KO, Fig. 1 lane 9). To examine the distribution of PUF5, we expressed a C-terminally myc-tagged version from a tetracycline-inducible promoter. This was clearly detectable with the PUF5 antibody (Fig. 1, lane 11) so it must have been considerably over-expressed relative to the wild-type cells. A minor faster-migrating band may be a degradation product.

**Figure 2:**
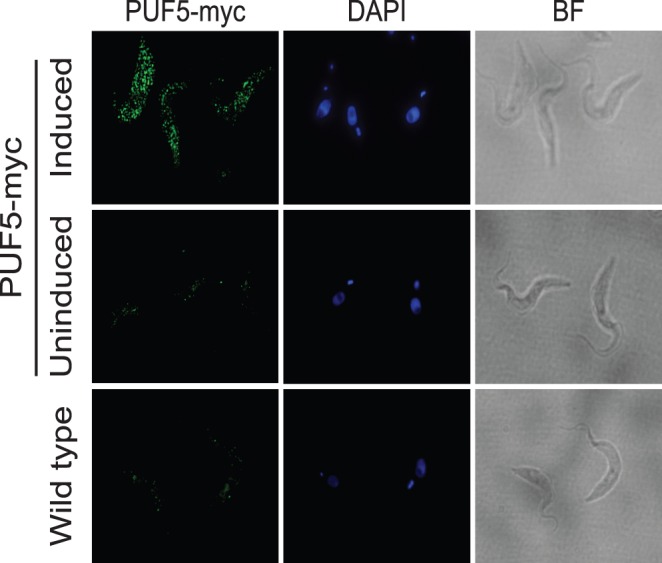
PUF5-myc is in the cytoplasm. An aliquot of cells expressing C-terminally myc tagged PUF5 (see Fig. 1) was used to check the localization of the protein in procyclic trypanosomes. Wild-type cells were used as the negative control. BF refers to bright field. DAPI was used to stain the nucleus and kinetoplast.

By immunofluorescence, we found that the myc-tagged protein was mainly in the cytoplasm (Fig. 2), forming a somewhat speckled pattern. The significance of the speckling is not clear: it may be real but could also be caused by over-expression or the tag. We also used the affinity purified PUF5 antibody for immunofluorescence but we could see no difference between wild-type and knock-out procyclic forms, presumably because the normal amount of PUF5 is too low for detection.

5×10^4^ molecules of PUF5 would be 2.5 molecules per mRNA [44]. Procyclic forms inducibly expressing about 10^5^ molecules of PUF5-myc/cell (Figure 1B, lanes 4, 5, 8 and 9) grew normally (not shown). 5×10^4^ molecules per cell, distributed throughout the cytoplasm, is a concentration of roughly 1.5 pM. The dissociation constants (Kd) of PUF proteins’ binding to their target RNA sequences generally fall in the range of 1–50 nM [2], [45], [46], [47]. Even if PUF5 binds to its targets with a Kd of 1 nM, to bind 50% of the best RNA targets, at least a million molecules per cell would be required. Therefore, if such binding does occur, it must be aided by either local concentration of the protein, or extensive cooperative interactions. Indeed, it has been shown that the interaction of FBF proteins with RNA can be influenced by interactions with a second RNA-binding protein, CPEB [48], and the action of Puf5p in repressing translation of *HO* mRNA requires the presence of poly(A) binding protein Pab1p [49].

### PUF5 Depletion does not Affect growth of Cultured Trypanosomes

**Figure 3:**
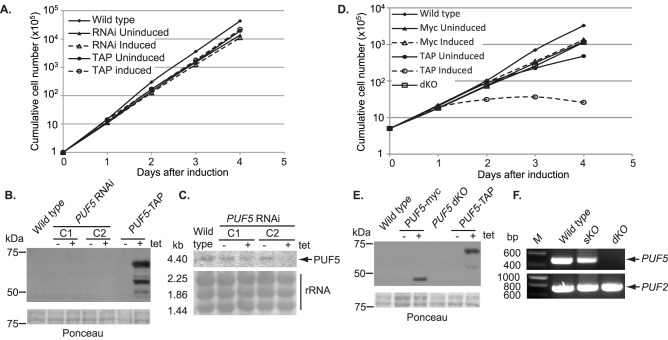
PUF5 is not essential for survival of PC trypanosomes. A. Cumulative growth curves of the bloodstream cells showing no difference in proliferation after *PUF5* RNAi or ectopic expression of C-terminally TAP-tagged PUF5. B. Western blot probed with anti-PUF5. C1 and C2 are different RNAi clones; their growth was indistinguishable. Ponceau S is the loading control. 5×10^6^cells loaded per lane. C. Northern blot for expression of *PUF5* RNA. Methylene-blue stained ribosomal RNA bands served as loading controls. D. Cumulative growth curves of procyclic cells ectopically expressing C-terminally myc tagged PUF5 or C-terminally TAP tagged PUF5. Results for procyclic *PUF5* double knockout cells (dKO) are also shown. E. Western blot to detect ectopic PUF5 expression, details as in (B). F. PCR amplification of a 443 bp fragment of the *PUF5* ORF in DNA from procyclic cells. A 708 bp fragment of *TbPUF2* ORF served as positive control. sKO: single knockout: dKO: double knock-out.

To investigate the role of PUF5, we first depleted it by RNAi. There was no effect in either life-cycle stage: Fig. 3A shows the growth of bloodstream forms. Depletion of the protein could not be measured because it was not detected (Fig. 3B) but the RNA was clearly decreased (Fig. 3C). We then knocked the gene out in PC forms. Again no growth defect was seen (Fig. 3D) although the gene was clearly absent (Fig. 3E, F). We were unable to obtain *PUF5* double-knockout bloodstream-form trypanosomes, which could either be a technical failure, or could indicate that they require a low amount of PUF5 for growth. Expression of myc-tagged PUF5 in procyclic forms did not affect growth (Fig. 3D).

**Figure 4:**
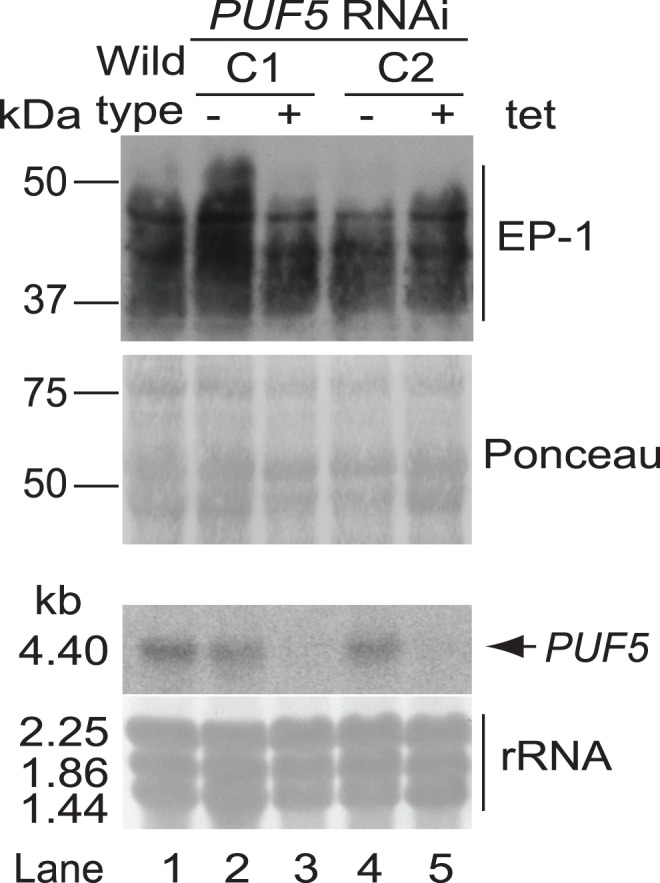
*PUF5* knockdown does not affect differentiation of trypanosomes from the bloodstream to the procyclic form. Differentiation competent trypanosomes with *PUF5* RNAi were induced to differentiate into procyclic forms. This Figure shows a Western blot (top two panels) and a Northern blot (bottom two panels) for wild-type trypanosomes and two different RNAi clones, C1 and C2, after growth had resumed, but no difference was seen at any stage during the differentiation. The Western blot shows expression of EP procyclin and the Northern blot shows successful knockdown of *PUF5.* Ponceau-stained protein and methylene-blue stained rRNA serve as loading controls.

We also tested whether PUF5 was required for differentiation. We generated differentiation-competent trypanosomes with *PUF5* RNAi, induced differentiation, and examined expression of the procyclic marker EP procyclin. There was no difference in EP procyclin expression at any point (e.g. Fig. 4) and no effect on the ability of the cells to resume growth in procyclic-form medium (not shown).

### Expression of *PUF5* Bearing a 21 kDa Tag Inhibits Trypanosome Growth

To look for interaction partners of PUF5, we inducibly expressed a version with a C-terminal tandem affinity purification (TAP) tag (Fig. 3B). Unfortunately, however, PUF5-TAP expression in PC cells led to growth arrest. Even the uninduced cells grew rather slowly, which might be because of leakiness of the tet-inducible promoter.

In these experiments, PUF5-TAP expression inhibited growth, whereas PUF5-myc expression did not. This is unlikely to be due to the difference in expression level. The signal from PUF5-TAP was only slightly stronger than that for PUF5-myc in Western blots (Fig. 3E), although the TAP tag binds non-specifically to IgG. However, in some other previous experiments we also observed growth inhibition upon expression of PUF5-myc and even (once) with PUF5 with no tag. A simple explanation would be that C-terminally tagged PUF5 is non-functional, and is competing with native PUF5. This does not, however, explain the growth effect since PUF5 itself is not essential. Perhaps the tag is irrelevant. Instead, there may be some mRNAs with regulatory sequences that are usually bound to, and regulated by, some other protein, but also have weaker affinity for PUF5. Over-expression of PUF5 would allow PUF5 to compete with the other RNA-binding protein and to disrupt regulation. Competition of PUF5 for binding to another protein that is present in limiting amounts is another possibility.

### Tagged PUF5 Binds to RNA in Procyclic Form Trypanosomes

**Figure 5:**
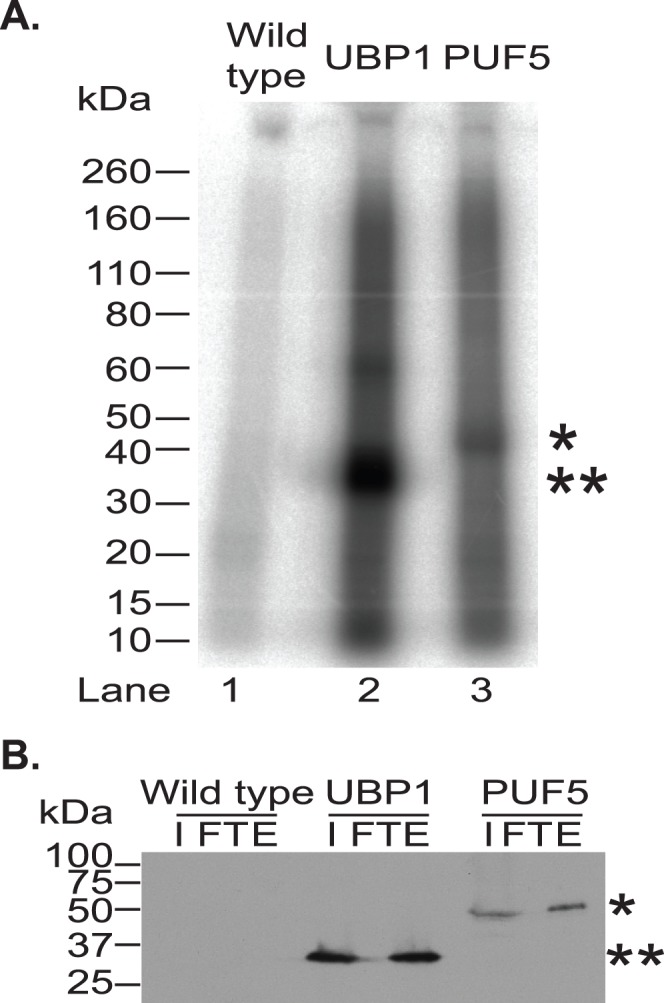
PUF5-myc binds to RNA. A. Cells expressing myc-tagged PUF5-myc or UBP1-myc were UV-irradiated, protein was immunoprecipitated with anti-myc antibody, and the bound RNA was radioactively end-labelled. Samples were run on a denaturing SDS-PAGE and RNA detected by phosphorimaging. ‘*’ indicates the band corresponding to the ^32^P labelled PUF5 bound RNAs while ‘**’ indicates the position of UBP1-bound RNAs. Wild type refers to the procyclic cell lines without any tagged protein. B. 10% of each sample was taken for western blot analysis using anti-myc antibody. ‘I’ refers to the input (cell lysate), ‘FT’ (flow through) refers to the unbound fraction and ‘E’ (eluate) refers to the bound fraction. Each sample represents approximately the same number of input trypanosomes.

To determine whether PUF5 can bind to RNA in vivo, we used procyclic trypanosomes expressing PUF5-myc. They were subjected to UV irradiation in order to cross-link proteins to RNA. After RNase treatment and anti-myc immunoprecipitation, the RNA associated with the protein was ^32^P labelled by kinase treatment, before separation by SDS-PAGE. The use of a denaturing gel during this procedure guarantees that any detected RNA was bound in the live cells, rather than associating after cell lysis. UBP1-myc [50] was included as a positive control, giving a clear radioactive band that ran at around 35 kDa (Fig. 5A, lane 2), whereas use of cells with no myc-tagged protein resulted in a smear without bands. Precipitation of PUF5-myc resulted in a band running around 45 kDa, roughly the expected size (Fig. 5A, lane 3 and Fig. 5B). The signal corresponding to the bound RNAs was, however, only slightly higher than background. This could reflect lower abundance of PUF5-myc than UBP1-myc, or binding of RNA by a smaller proportion of the PUF5-myc molecules. The signal was so weak that no further attempts were made to purify PUF5-associated RNAs.

## Conclusions

Our results showed that PUF5 is not essential for growth of procyclic trypanosomes in culture, and may also not be required in bloodstream forms. Knock-down also had no observable effect on the ability of trypanosomes to differentiate from the bloodstream to the procyclic form. Nevertheless, the gene is conserved in trypanosomes and Leishmanias, so it is unlikely to be functionless. The cytoplasmic trypanosome PUF proteins may have redundant functions, as in yeast [13], [14], *C.elegans* [15] and humans [16], or they might be required only under specific environmental conditions [12]. There is also a possibility that PUF5 could have a role in Tsetse life-cycle stages that were not analysed here, as described for RBP6 [51]. In this context, it would be interesting to know how much PUF5 is present in epimastigotes and metacyclic trypomastigotes in Tsetse flies. Our antibody would be useful for this.

## Supporting Information

File S1 **Contains:** Table S1: Plasmids used and made for this study. Table S2: Oligonucleotides used in this study: Restriction sites are underlined and hybridizing parts of the primers are in upper case
(PDF)
