# Supplementary material for: The Trypanosome Pumilio Domain Protein PUF5
Source: PLoS One. 2013 Oct 22;8(10):e77371. doi: 10.1371/journal.pone.0077371 (PMC3805580; doi:10.1371/journal.pone.0077371)
Supplement: File S1 — Contains: Table S1: Plasmids used and made for this study. Table S2: Oligonucleotides used in this study: Restriction sites are underlined and hybridizing parts of the primers are in upper case (PDF) [file pone.0077371.s001.pdf]

Table S1

Plasmids used in this study.

Plasmid	Description
pHD918	C-terminal TAP tag vector for inducible expression [31]
pHD2176	pHD918 + PUF5 ORF (*Hind*III/*Hpa*I)
pHD1700	C-terminal myc tag vector for inducible expression [32]
pHD1788	pHD1700 + PUF5 ORF (*Hind*III/*Hpa*I)
PHD1652	pHD1621 + PUF5-RNAi (*Xho*I/*Bam*HI) [26]
p2T7[TA]-blu	Antisense RNAi vector with two opposing T7 promoters (Blue/White selection) [33]
pHD2177	p2T7[TA]-blu + PUF5-RNAi (*Xho*I/*Bam*HI)
pHD1748	Knockout vector having blasticidin resistance ORF + Polylinker
pHD2385	pHD1748 + PUF5_5' (*Not*I/*Eco*RI) and 3' (*Xho*I/*Apa*I) UTRs
pHD1747	Knockout vector having puromycin resistance ORF + Polylinker
pHD2179	pHD1747 + PUF5_5' (*Apa*I/*Xho*I) and 3' (*Eco*RI/*Bam*HI) UTRs
pHD1746	pQTEV based vector for His tagging and expression in bacteria
pHD2153	pHD1746 + PUF5 ORF
pHD1146	Stem loop RNAi vector [34]
pHD2178	pHD1146 + PUF5 –RNAi (stem loop)

Table S2

Oligonucleotides used in this study. Restriction sites are underlined and hybridizing parts of the primers are in upper case

CZ number	Description	Sequence	Restriction site
CZ2259	PUF5ORF_Fw	gatcaagcttATGCTTCGTAGGGGTG	*Hind*III
CZ2261	PUF5ORF(*Hpa*I, -stop)_Rev	gatcgttaacCTCACCGACTGCCCCG	*Hpa*I
CZ2262	PUF5 RNAi_Fw	CTTGCTGTGAGTTCGCCATA	
CZ2263	PUF5 RNAi_Rev	TGACGGGATCACACACTGTT	
CZ2749	PUF5_5'UTR_Fw	gagcggccgcTGCAGCTCCTCCCTAGTGTT	*Not*I
CZ2750	PUF5_5'UTR_Rev	gagaattcTGTGTCGCAAAGCCAGTAAG	*Eco*RI
CZ2755	PUF5_3'UTR_Fw	gactcgagTCTTCCCACAAGTTCACACG	*Xho*I
CZ2732	PUF5_3'UTR_Rev	gagggcccCGCTCCTTTCATGATCAACC	*Apa*I
CZ3944	PUF5_5'UTR_Fw II	atgcgggcccAAGTGTGTGGGTGTTTGTGC	*Apa*I
CZ3945	PUF5_5'UTR_Rev II	atgcctcgagACCTACCACAACCGACAAGG	*Xho*I
CZ3946	PUF5_3'UTR_Fw II	atgcgaattcTCTTCCCACAAGTTCACACG	*Eco*RI
CZ3947	PUF5_3'UTR_Rev II	atgcggatccTCTTATCGCTTGCCTTCGTT	*Bam*HI
CZ3956	PUF5RNAi_Fw II	gagaagatct*gcatgc*AGCTCTCAACGTTCCACAA	*Bgl*II, *Sph*I
CZ3957	PUF5RNAi_Rev II	cggaattc*gtcgac*CCGACACTAACAAGTTGCGA	*Eco*RI, *Sal*I
CZ4024	PUF2YFP_Fw	gatcggtaccCAGATAAGGCCGCACCTC	*Kpn*I
CZ4025	PUF2YFP_Rev	gatcggatccCAGCGTTGGCATGCAGGA	*Bam*HI
